# Receptor C_k_-dependent signaling regulates hTERT gene transcription

**DOI:** 10.1186/1471-2121-7-2

**Published:** 2006-01-12

**Authors:** Kavleen Sikand, Deepak Kaul, Neelam Varma

**Affiliations:** 1 Department of Experimental Medicine & Biotechnology, Postgraduate Institute of Medical Education and Research, Chandigarh – 160 012, India; 2 Department of Haematology, Postgraduate Institute of Medical Education and Research, Chandigarh – 160 012, India

## Abstract

**Background:**

Available evidence suggests that the regulation of telomerase activity primarily depends on the transcriptional control of the human telomerase reverse transcriptase (hTERT) gene. Although several activators and repressors of hTERT gene transcription have been identified, the exact mechanism by which hTERT transcription is repressed in normal cells and activated in cancer cells remains largely unknown. In an attempt to identify possible novel mechanisms involved in the regulation of hTERT transcription, the present study examined the role of Receptor C_k_, a cell surface receptor specific for cholesterol, in the transcription of hTERT gene in normal human peripheral blood mononuclear cells.

**Results:**

Activated Receptor C_k _was found to down-regulate hTERT mRNA expression by repressing the transcription of c-myc gene. Receptor C_k_-dependent signaling was also found to down-regulate the mRNA expression of the gene coding for the ligand inducible transcription factor, peroxisome proliferator-activated receptor γ (PPARγ). The ligand activation of PPARγ resulted in the down-regulation of c-myc and hTERT mRNA expression. By using specific activator and inhibitor of protein kinase C (PKC), it was demonstrated that Receptor C_k _dependent down-regulation of hTERT gene transcription involved inhibition of PKC. In addition, 25-hydroxycholesterol was found to contribute to the transcriptional regulation of hTERT gene.

**Conclusion:**

Taken together, the findings of this study present evidence for a molecular link between cholesterol-activated Receptor C_k _and hTERT transcription, and provide new insights into the regulation of hTERT expression in normal human peripheral blood mononuclear cells.

## Background

Telomeres are specialized DNA-protein complexes that serve as protective caps of linear eukaryotic chromosomal ends and are essential for chromosomal stability [1]. The replication of telomeres during cell division poses a special problem for the cells because the conventional DNA polymerases are unable to completely replicate the chromosome ends. Most eukaryotic cells employ the enzyme telomerase for the maintenance of telomeres. Telomerase is a RNA-dependent DNA polymerase that synthesizes telomeric DNA sequences and almost universally provides the molecular basis for unlimited proliferative potential. The telomerase activity has been found to be absent in most normal somatic cells but present in over 90% of cancerous cells and in vitro immortalized cells [2,3]. Telomerase is also expressed in germ line cells and in a small number of normal somatic cells that possess high turnover capability, such as, haematopoietic stem cells, peripheral blood lymphocytes, skin keratinocytes, intestinal crypt cells etc. [4]. Due to the absence of telomerase in most normal somatic cells, telomeres in these cells, shorten with progressive cell division in vitro and with increased age in vivo [5,6]. This telomere shortening has been proposed to act as a mitotic clock, which counts the number of divisions undergone by a cell and triggers cellular senescence at a critically short telomere length. The maintenance of telomere length by telomerase is thus essential for long-term cell proliferation and cell immortalization. The mechanisms involved in the regulation of telomerase activity are of special interest since the modulation of telomerase activity can be used to alter cellular life span and thus, can be exploited in anti-cancer and anti-ageing therapies.

The human telomerase consists of two essential components: (i) the human telomerase RNA component (hTR or hTERC) and (ii) the catalytic component, known as the human telomerase reverse transcriptase (hTERT) [7]. Several other protein subunits associate with these core components to form the telomerase holoenzyme that synthesizes telomeric DNA [7]. Among the multiple components of human telomerase, only the catalytic subunit, hTERT seems to be the key determinant of telomerase activity. While the expression of hTERT is repressed in normal somatic cells (telomerase negative) and is upregulated in immortal cells (telomerase positive), the other components of telomerase holoenzyme are usually expressed ubiquitously in both telomerase positive and telomerase negative cells [7]. Although various steps at post-transcriptional and post-translational levels have been found to modulate telomerase activity, substantial experimental data have established that the transcriptional control of hTERT gene is the major contributor to the regulation of telomerase activity in most cell types [7]. Binding sites for several transcription factors have been identified on the promoter of hTERT gene. Transcription factors such as c-myc, Sp1, nuclear factor-κB (NF-κB) and upstream stimulatory factors (USF) have been shown to upregulate hTERT transcription whereas transcription factors like Mad1, Wilms' tumor 1 (WT1) tumor suppressor protein, E2F, p53 and myeloid cell-specific zinc finger protein-2 (MZF-2) have been found to down-regulate hTERT transcription [7-9]. Bcl-2, the oncoprotein involved in the inhibition of cellular apoptosis has also been reported to modulate telomerase activity [10,11]. Several studies have implicated sex steroid hormones (estrogen, progesterone and androgens) in the regulation of hTERT transcription [7]. Some human chromosomes (chromosome 3, 4, 6, 7, 10 and 17) have been found to potentially contain transcriptional repressors of hTERT [12]. The hTERT gene is also a target site for viruses frequently associated with human cancers, such as human papillomavirus and hepatitis B virus [12]. Further, some studies suggest that methylation of the promoter of hTERT gene may contribute to the repression of hTERT gene transcription in some cell types [12]. There is also evidence to suggest that chromatin remodeling by various transcription factors may be involved in the regulation of hTERT gene transcription [12]. Although the enormous amount of research has identified numerous protein factors as putative hTERT regulators, the exact mechanisms involved in the regulation of hTERT transcription are still far from being fully established. It is likely that as yet unidentified factors are important players in the control of activation and repression of hTERT transcription.

In this context, Receptor C_k_, a cell-surface receptor specific for the cholesterol moiety in lipoprotein particles [13], may be a good candidate for the regulation of hTERT transcription. Receptor C_k _has been shown to initiate a signaling pathway that controls the expression of several genes involved in cell cycle, such as, the genes encoding cyclin D, Bcl-2, p27, c-myc and c-fos [14]. Out of these Receptor C_k _target genes, the c-myc and Bcl-2 gene products are known modulators of hTERT transcription and telomerase activity. Further, investigations have revealed that Receptor C_k _dependent signaling is defective in various leukemic cell lines/patients and also in tumors of the central nervous system [15,16]. The present study was thus, designed to explore the effect, if any, of Receptor C_k _dependent signaling on hTERT transcription. We also sought to identify the various transcription factors involved in the above process. Besides the transcription factors c-myc and Bcl-2, which were obvious candidates for the potential link between Receptor C_k _and hTERT expression, we also examined cyclin D and peroxisome proliferator-activated receptor γ (PPARγ). D-type cyclins are rate-limiting controllers of G1 phase progression in mammalian cells. Overexpression of these cyclins has been detected in several types of cancers and an oncogenic role for them has been suggested [17]. Chromosomal translocations involving cyclin D1 locus have been reported in different tumor types, such as, carcinoma of the breast, oesophagus, stomach, bladder and liver, and in squamous carcinomas of the head and neck [18]. PPARγ, a ligand activated transcription factor of the nuclear hormone receptor family, was initially characterized as a regulator of adipogenesis and glucose homeostasis [19]. New investigations have revealed that PPARγ also regulates cell proliferation/differentiation pathways and the expression of many genes involved in carcinogenesis [19,20]. Given their involvement in tumorigenesis, both cyclin D and PPARγ are potential candidates for the regulation of hTERT transcription.

The majority of studies have evaluated the regulation of hTERT and telomerase in tumor models. There is a relative paucity of information on this front in normal cells. Hence, the cellular models employed in the present study were the normal human peripheral blood mononuclear cells (PBMCs), which are known to express hTERT and telomerase in a highly regulated manner. Our results revealed a novel mechanism for the regulation of hTERT transcription controlled by cholesterol activated Receptor C_k_.

## Results

### Involvement of Receptor C_k _in the transcription of hTERT, c-myc and PPARγ genes

**Figure 1:**
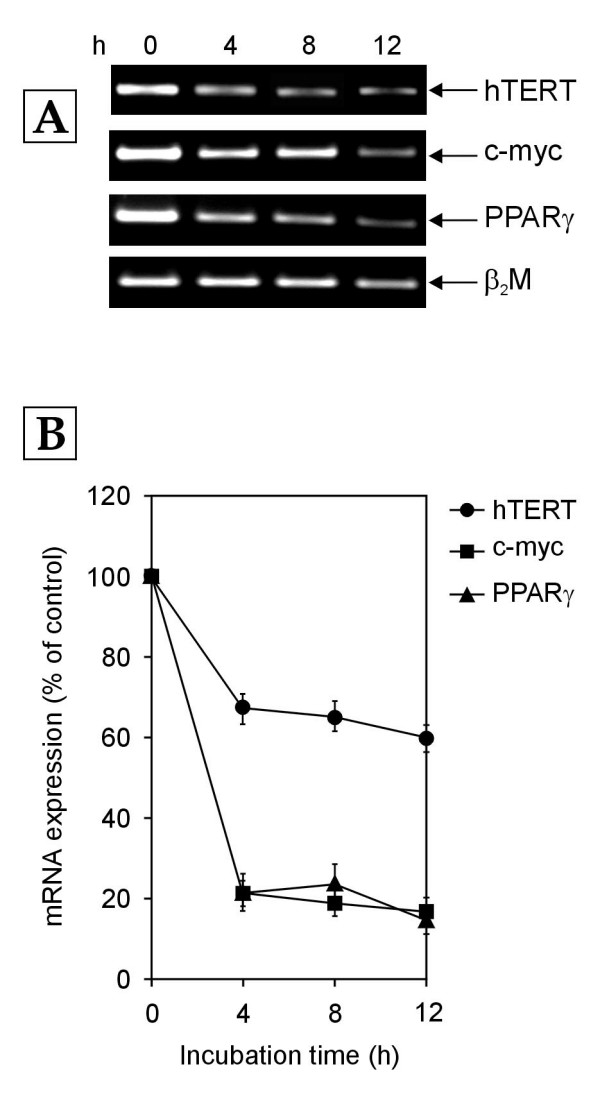
**Time course of hTERT, c-myc and PPARγ mRNA expression in normal human PBMCs exposed to growth medium enriched with 10% NHS**. The cells were harvested at indicated time points (h-hours) and subjected to RNA extraction followed by RT-PCR. (A) Representative agarose gel photographs showing ethidium bromide stained RT-PCR products of hTERT, c-myc, PPARγ and β_2_M genes. (B) The signal intensities of RT-PCR products shown in panel A were measured using SCION IMAGE analysis software. The relative levels of hTERT, c-myc and PPARγ mRNA expression in each lane were determined by normalizing their individual band intensity to β_2_M band intensity. The mRNA expression of each gene at 4, 8 and 12 h is plotted as percentage of that in control cells (i.e. cells harvested at 0 h). Each data point in the graph represents mean ± standard deviation (SD) of three separate experiments.

**Figure 2:**
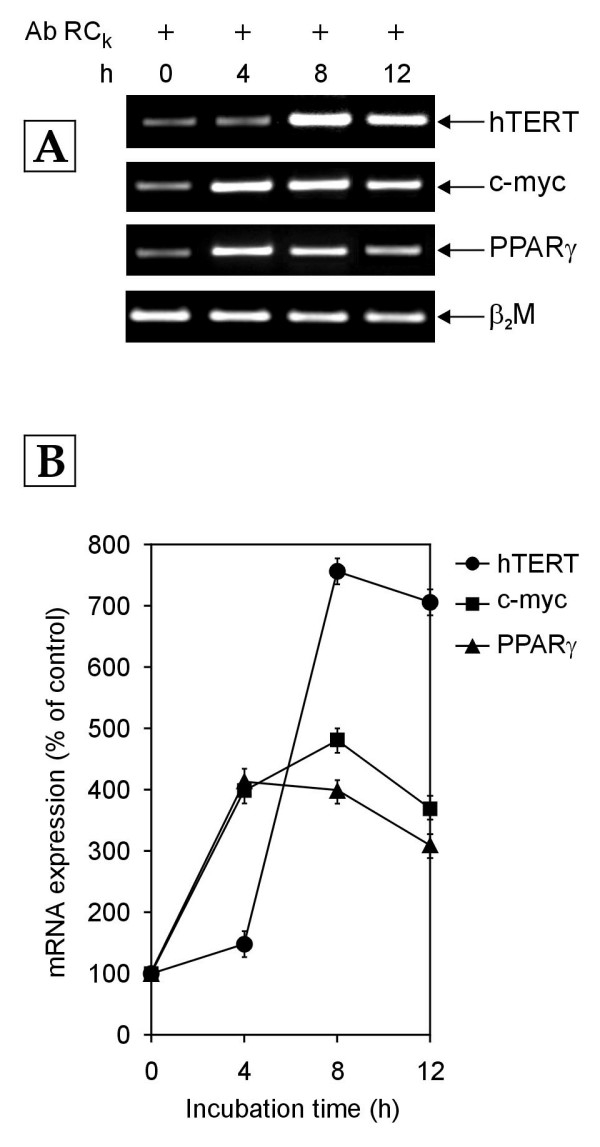
**The mRNA expression of hTERT, c-myc and PPARγ genes in normal human PBMCs treated with growth medium containing 10% NHS along with antibody against Receptor C_k _(Ab RC_k_)**. Total cellular RNA was extracted at indicated time points (h-hours) and analyzed by RT-PCR. (A) Representative agarose gel photographs showing ethidium bromide stained RT-PCR products of hTERT, c-myc, PPARγ and β_2_M genes. (B) The signal intensities of these RT-PCR products were measured using SCION IMAGE analysis software. The mRNA expression was determined by normalizing the band intensity of target mRNA (hTERT, c-myc and PPARγ) to β_2_M band intensity. Data are expressed as percentage of control (cells harvested at 0 h). Each data point in the graph represents mean ± SD of three independent experiments.

In order to determine whether cholesterol-activated Receptor C_k _regulates the transcription of hTERT, c-myc and PPARγ genes in normal human PBMCs, the cells were cultured in growth medium enriched with 10% normal human serum (NHS) in the presence and absence of a polyclonal monospecific antibody against Receptor C_k_. These cells were harvested at various time points and the cellular mRNA levels of hTERT, c-myc and PPARγ genes were measured by RT-PCR. In the cells exposed to growth medium containing 10% NHS alone, Receptor C_k _was activated by the cholesterol moiety of low density lipoprotein (LDL) supplied by 10% NHS and a decrease in the mRNA levels of hTERT, c-myc and PPARγ genes over 12 hours was observed (Figure 1). This decrease was abolished in the presence of the antibody against Receptor C_k_, which blocked Receptor C_k _activation. As shown in Figure 2, addition of antibody against Receptor C_k _resulted in the upregulation of mRNA expression of hTERT, c-myc and PPARγ genes. Taken together, these results indicate that Receptor C_k _dependent signaling regulates the transcription of hTERT, c-myc and PPARγ genes.

**Figure 3:**
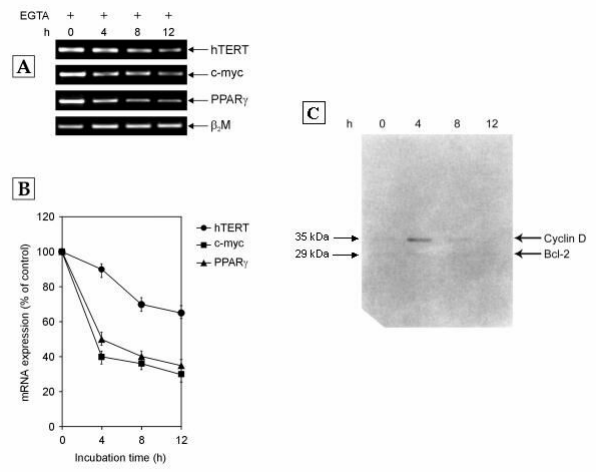
**The expression of hTERT, c-myc, PPARγ, cyclin D and bcl-2 genes over 12 h (hours) in normal human PBMCs treated with growth medium containing 10% NHS and 2.5 mM EGTA**. The cells were harvested at indicated time points and subjected to RT-PCR and western blotting. (A) Representative agarose gel photographs showing ethidium bromide stained RT-PCR products of hTERT, c-myc, PPARγ and β_2_M genes. (B) Graph showing the changes in mRNA levels of hTERT, c-myc and PPARγ genes with increasing time after EGTA treatment. The relative levels of hTERT, c-myc and PPARγ mRNA expression were determined by measuring their band intensities using SCION IMAGE analysis software and normalizing to β_2_M band intensity. Data are expressed as percentage of control (0 h). Each data point represents mean ± SD of three independent experiments. (C) Western blot analysis of expression of cyclin D and bcl-2 proteins. Equal amount of protein was used for the four samples. The western blot shown is a representative of three independent experiments.

Since two receptors for LDL, namely apolipoprotein B specific 'low density lipoprotein receptor (LDLR)' and LDL-cholesterol specific 'Receptor C_k_' are present on cells, we wanted to confirm that the observed effect on the transcription of hTERT, c-myc and PPARγ genes was not due to LDLR. The possible involvement of LDLR was ruled out by culturing normal human PBMCs in growth medium enriched with 10% NHS in the presence of 2.5 mM ethyleneglycol-bis-(β-aminoethylether)-N,N,N',N' -tetraacetic acid (EGTA). EGTA is a known blocker of the interaction of LDL with LDLR [21]. Hence, in the presence of EGTA, LDLR pathway is shut off while Receptor C_k _signaling is functional. The results obtained after EGTA addition (Figure 3A &3B) were similar to those observed when PBMCs were exposed to growth medium enriched with 10% NHS without EGTA (Figure 1) thereby showing that the blockage of LDLR pathway had no effect upon the transcription of these genes. Hence, the cholesterol specific Receptor C_k _and not LDLR was responsible for the transcriptional regulation of hTERT, c-myc and PPARγ genes.

### Role of Bcl-2 and cyclin D in Receptor C_k _dependent regulation of hTERT gene transcription

Keeping in view the established link among Receptor C_k_, Bcl-2 and cyclin D [22], it was reasonable to speculate that cyclin D and Bcl-2 could mediate the effect of Receptor C_k _on hTERT gene transcription. However, on comparing the expression pattern of cyclin D and Bcl-2 proteins (Figure 3C) with the hTERT mRNA expression in human normal PBMCs exposed to growth medium containing 10% NHS and 2.5 mM EGTA (Figure 3B), no correlation was found between cyclin D expression and hTERT transcription or between Bcl-2 expression and hTERT transcription. While cyclin D protein was expressed at only 4 hours and Bcl-2 protein was not expressed at all (Figure 3C), the level of hTERT mRNA gradually decreased over 12 hours (Figure 3B). Hence, it was inferred that cyclin D and Bcl-2 might not play any role in the regulation of hTERT transcription in normal human PBMCs.

### Role of c-myc in Receptor C_k _dependent regulation of hTERT gene transcription

**Figure 4:**
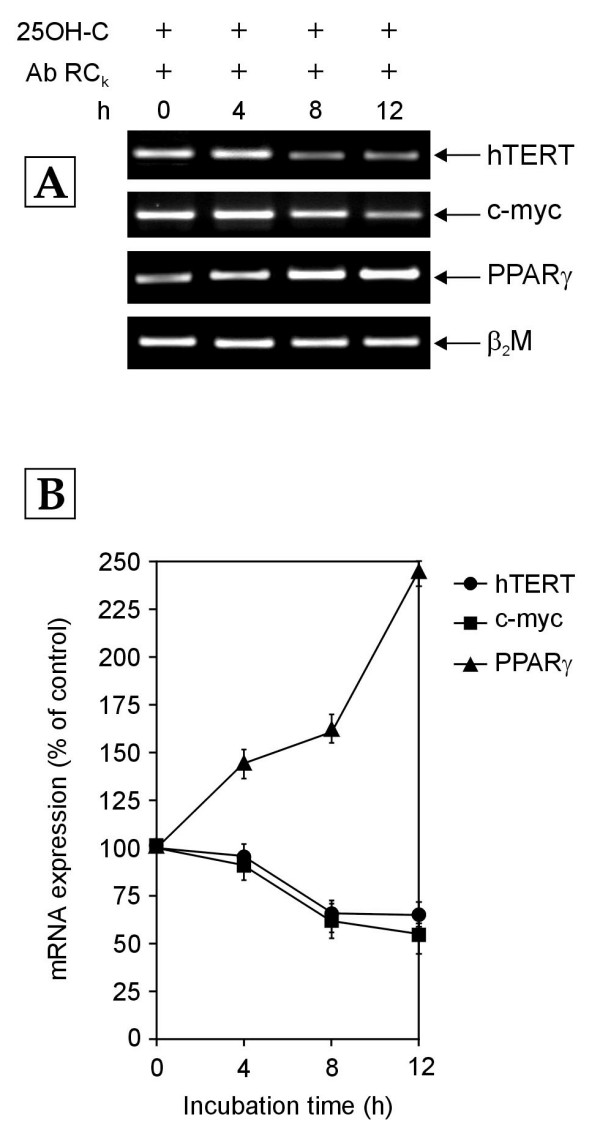
**The hTERT, c-myc and PPARγ mRNA levels over 12 h (hours) in normal human PBMCs exposed to growth medium containing 10% NHS along with antibody against receptor C_k _(Ab RC_k_) and 25-hydroxycholesterol (25OH-C; 1 μM)**. Total cellular RNA was extracted at indicated time points and amplified by RT- PCR. (A) Representative agarose gel photographs showing ethidium bromide stained RT-PCR products of hTERT, c-myc, PPARγ and β_2_M genes. (B) The signal intensities of these RT-PCR products were measured using SCION IMAGE analysis software. The relative levels of hTERT, c-myc and PPARγ mRNA expression were determined by normalizing their individual band intensity to β_2_M band intensity. The mRNA expression of each gene at 4, 8 and 12 h is plotted as percentage of that in control cells (cells harvested at 0 h). Each data point represents mean ± SD for the combined results of three separate experiments.

Substantial data have implicated c-myc in the upregulation of hTERT transcription [7]. In order to determine whether or not c-myc mediates the Receptor C_k _dependent down-regulation of hTERT gene transcription, normal human PBMCs cultured in medium containing 10% NHS were exposed to antibody against Receptor C_k _along with 1 μM of 25-hydroxycholesterol (25OH-C), which is a negative regulator of c-myc mRNA and protein expression [23]. As expected, c-myc mRNA levels declined gradually over 12 hours due to the presence of 25OH-C (Figure 4). A similar decrease was observed in hTERT mRNA expression (Figure 4), thus proving that Receptor C_k _regulates hTERT gene transcription indirectly by controlling the transcription of c-myc gene. The PPARγ mRNA levels were not affected by the addition of 25OH-C. In both the cases – PBMCs exposed only to antibody against Receptor C_k _(Figure 2) and PBMCs exposed to antibody against Receptor C_k _along with 25OH-C (Figure 4) – the PPARγ transcript levels increased over 12 hours, demonstrating the high specificity of the inhibitory effect of 25OH-C on c-myc mRNA expression.

### Effect of 25OH-C on the Receptor C_k _dependent transcriptional regulation of hTERT, c-myc and PPARγ genes

**Figure 5:**
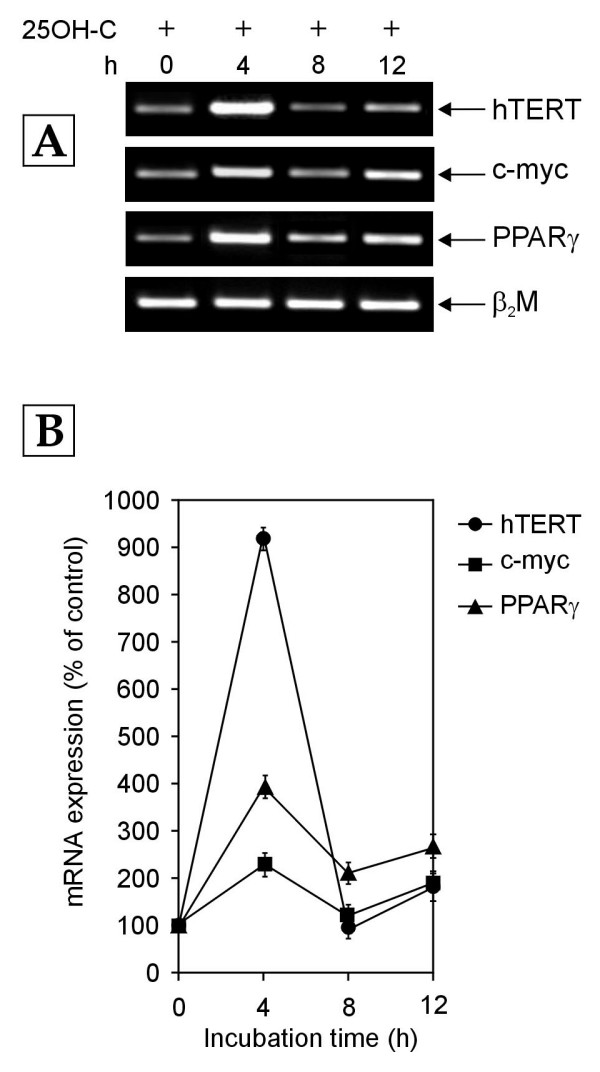
**RT-PCR analysis of expression of hTERT, c-myc and PPARγ mRNA in normal human PBMCs treated with growth medium containing 10% NHS and 25-hydroxycholesterol (25OH-C; 1 μM) for 0–12 h (hours)**. (A) Representative agarose gel photographs showing ethidium bromide stained RT-PCR products of hTERT, c-myc, PPARγ and β_2_M genes. (B) Graph showing the changes in mRNA levels of hTERT, c-myc and PPARγ genes with increasing time, after 25OH-C treatment. The relative levels of hTERT, c-myc and PPARγ mRNA expression were determined by measuring their band intensities using SCION IMAGE analysis software and normalizing to β_2_M band intensity. Data are expressed as percentage of control (0 h). Each data point represents mean ± SD of three independent experiments.

As a control, the effect of 25OH-C was also investigated in PBMCs cultured in the absence of antibody against Receptor C_k_. The exposure of PBMCs to growth medium containing 10% NHS had resulted in a gradual decrease in the mRNA levels of c-myc, PPARγ and hTERT genes over 12 hours (Figure 1). Due to the negative regulation of the c-myc mRNA expression by 25OH-C, the addition of 25OH-C to this culture system was expected to further decrease c-myc and hTERT mRNA levels while not affecting the decline of PPARγ mRNA levels. However, surprisingly, the exposure of normal human PBMCs to growth medium containing 10% NHS along with 1 μM 25OH-C revealed a completely different scenario (Figure 5). Addition of 25OH-C transiently abolished the Receptor C_k _dependent transcriptional down-regulation of hTERT, c-myc and PPARγ genes observed in PBMCs cultured in growth medium containing 10% NHS alone (Figure 1) and exhibited a biphasic effect on the transcription of these genes (Figure 5). The hTERT transcript levels increased by about 9.3 fold as compared to the levels in control cells after 4 hours of 25OH-C treatment (Figure 5). These levels then decreased to the hTERT mRNA levels in control cells, at 8 hours and again increased slightly (about 1.7 fold) at 12 hours (Figure 5). The c-myc and PPARγ mRNA expression also followed a similar pattern of time-dependent increase and decrease though the fold increase was less as compared to that seen in hTERT mRNA expression (Figure 5). The observation that the addition of 25OH-C to PBMCs cultured in medium enriched with 10% NHS is able to disrupt the Receptor C_k _dependent down-regulation of hTERT, c-myc and PPARγ mRNA expression (Figures 1 &5) indicates that 25OH-C also contributes to the transcriptional regulation of these genes in normal human PBMCs. More importantly, the data suggest the possibility that the presence of an oxysterol along with LDL could influence and alter the transcriptional regulation of hTERT, c-myc and PPARγ genes by LDL-cholesterol activated Receptor C_k_.

### Link between PPARγ activation and hTERT gene transcription

**Figure 6:**
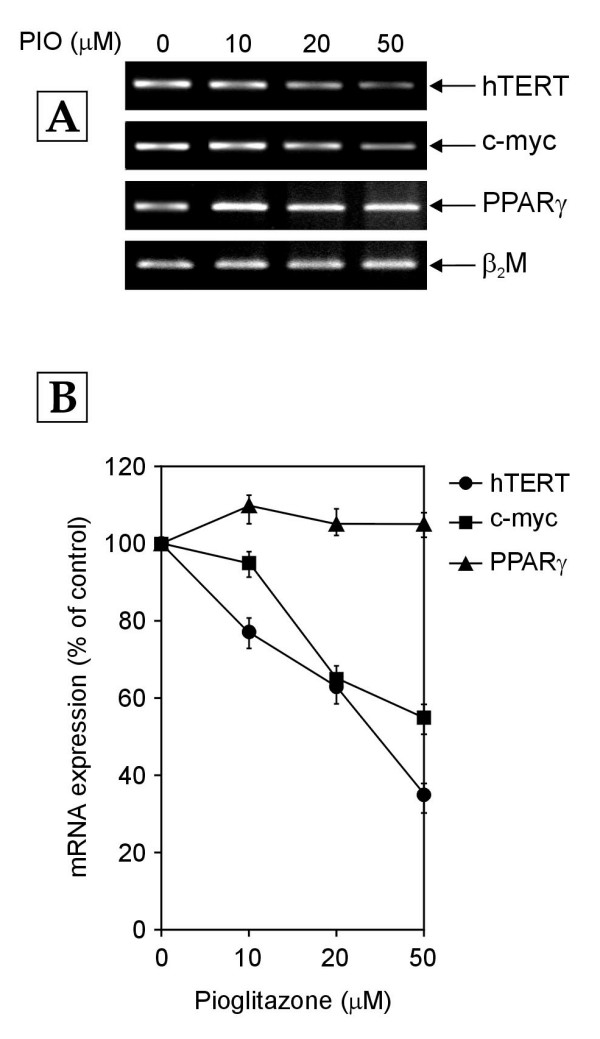
**Effect of pioglitazone (PIO) on the mRNA expression of hTERT, c-myc and PPARγ genes**. The normal human PBMCs were treated with the indicated concentrations of pioglitazone and with vehicle (DMSO) alone (0 μM pioglitazone, control) for 24 hours. Total cellular RNA was extracted from harvested cells and subjected to RT-PCR analysis. (A) Representative agarose gel photographs showing ethidium bromide stained RT-PCR products of hTERT, c-myc, PPARγ and β_2_M genes. (B) The signal intensities of RT-PCR products shown in panel A were measured using SCION IMAGE analysis software. The relative levels of hTERT, c-myc and PPARγ mRNA expression were determined by the ratio of their individual band intensity to β_2_M band intensity. The mRNA levels are plotted as percentage of those in control cells. Each data point in the graph represents mean ± SD of three separate experiments.

To evaluate the effect of PPARγ activation on the transcription of hTERT, c-myc and PPARγ genes, the normal human PBMCs were treated with different concentrations (0–50 μM) of pioglitazone, a PPARγ agonist [24] and mRNA expression of hTERT, c-myc and PPARγ genes was determined by RT-PCR after 24 hours of pioglitazone treatment. PPARγ mRNA levels remained largely unchanged with increasing concentrations of pioglitazone (Figure 6), showing that the agonist had no effect on PPARγ gene transcription. On the other hand, c-myc and hTERT mRNA expression exhibited a dose-dependent decrease (Figure 6), thus implicating the transcription factor, PPARγ in the regulation of the transcription of c-myc and hTERT genes.

**Figure 7:**
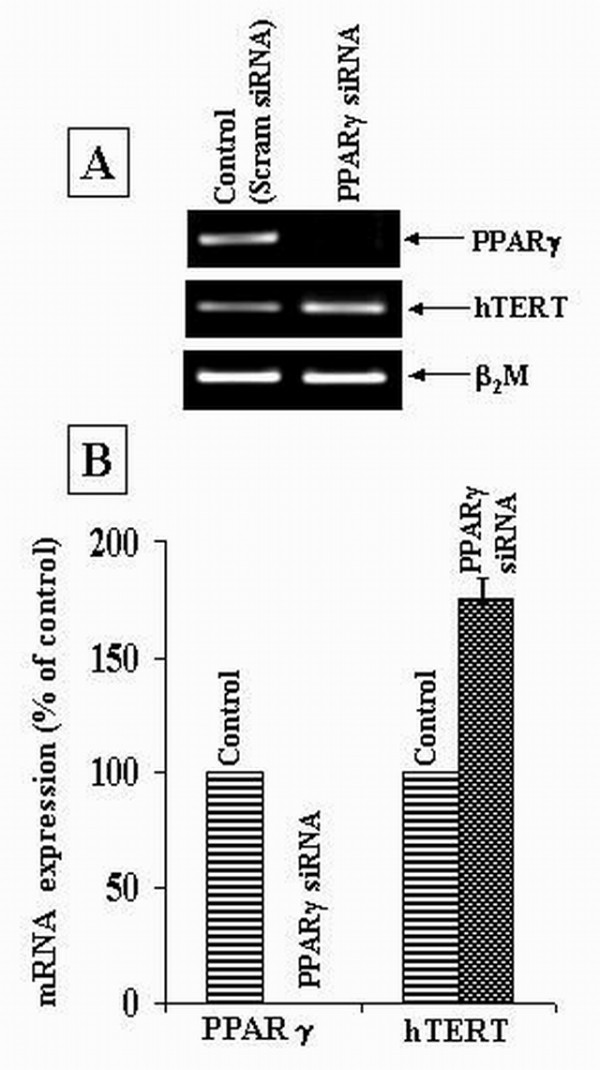
**Effect of PPARγ silencing on hTERT gene transcription**. Normal human PBMCs were transfected with scrambled siRNA (control scram siRNA) or with siRNA targeting PPARγ mRNA (PPARγ siRNA). These cells were cultured for 72 hours and then subjected to RNA extraction and RT-PCR analysis. (A) Representative agarose gel photographs showing ethidium bromide stained RT-PCR products of PPARγ, hTERT and β_2_M genes. (B) The signal intensities of RT-PCR products were measured using SCION IMAGE analysis software. The mRNA expression was determined by normalizing the band intensity of target mRNA (hTERT and PPARγ) to β_2_M band intensity. The hTERT and PPARγ mRNA levels in cells transfected with PPARγ siRNA are expressed as percentage of that in control (scram siRNA) cells. Each bar represents mean ± SD of experiments done in triplicate.

To further verify the link between PPARγ and hTERT gene transcription, we used PPARγ gene-specific small interfering RNA (siRNA) to knock down PPARγ mRNA expression in normal human PBMCs and determined the effect of PPARγ silencing on hTERT gene transcription. hTERT mRNA expression was found to increase in PPARγ knockout cells as compared to that in control cells (Figure 7), thereby indicating the role of PPARγ as a negative regulator of hTERT transcription.

### Role of PKC in Receptor C_k _dependent signaling

**Figure 8:**
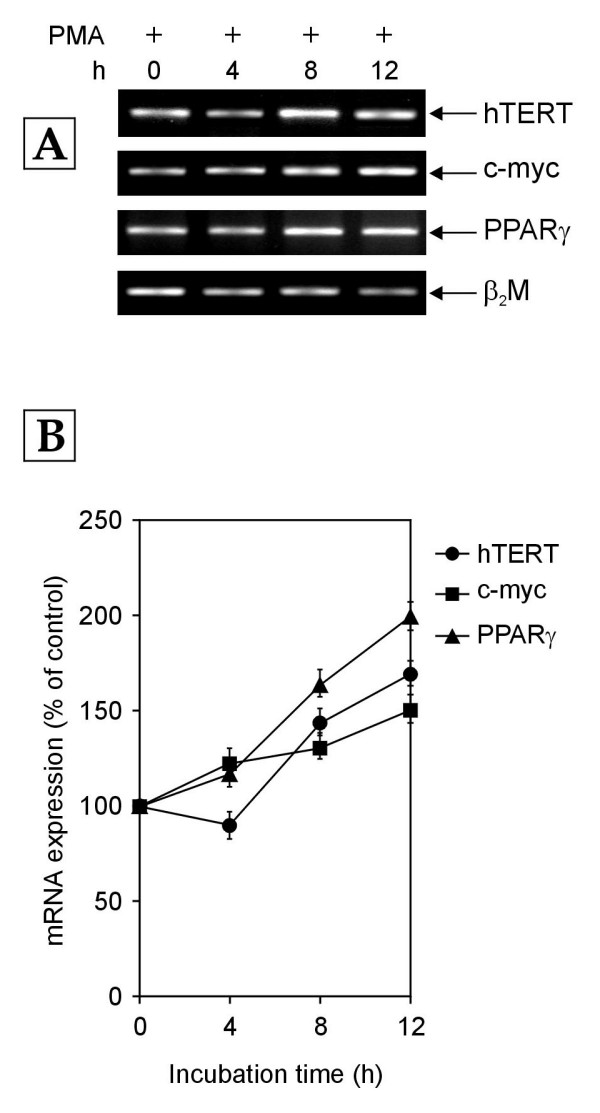
**Time course of hTERT, c-myc and PPARγ mRNA expression after treatment of normal human PBMCs with growth medium containing 10% NHS and PMA (100 nM)**. Total cellular RNA was extracted at indicated time points (h-hours) and analyzed by RT-PCR. (A) Representative agarose gel photographs showing ethidium bromide stained RT-PCR products of hTERT, c-myc, PPARγ and β_2_M genes. (B) The signal intensities of these RT-PCR products were quantified using SCION IMAGE analysis software. The relative levels of hTERT, c-myc and PPARγ mRNA expression were obtained by the ratio of their individual band intensity to β_2_M band intensity. The normalized mRNA expression of each gene at 4, 8 and 12 h is plotted as percentage of that in control cells (0 h). Each data point in the graph represents mean ± SD of three separate experiments.

Since previous studies have shown that Receptor C_k _activation leads to the generation of the second messenger, phosphatidic acid (PA) [22] and since PA can be dephosphorylated by the enzyme phosphatidic acid phosphohydrolase (PAP) to give rise to diacylglycerol (DAG), which is an endogenous activator of protein kinase C (PKC) [25,26], we hypothesized that PA, DAG and PKC may be involved in the Receptor C_k _dependent transcriptional regulation of c-myc, PPARγ and hTERT genes. In order to assess the involvement of PKC in the Receptor C_k _dependent regulation of the transcription of hTERT, c-myc and PPARγ genes, the normal human PBMCs were cultured in growth medium enriched with 10% NHS in the presence of 100 nM of phorbol 12-myristate 13-acetate (PMA), which is a known activator of PKC [27,28]. The addition of PMA abolished the decrease in hTERT, c-myc and PPARγ mRNA expression that was observed in normal human PBMCs cultured in medium enriched with 10% NHS alone (Figures 1 &8). The exposure of PBMCs to PMA resulted in a gradual increase in mRNA levels of hTERT, c-myc and PPARγ genes over 12 hours (Figure 8). The observation that PKC activation by PMA is able to reverse the Receptor C_k _dependent transcriptional down-regulation of hTERT, c-myc and PPARγ genes suggests that activated Receptor C_k _down-regulates the transcription of these genes by inhibiting PKC activation. Hence, PKC was implicated in the Receptor C_k _signaling pathway controlling the transcription of c-myc, PPARγ and hTERT genes.

**Figure 9:**
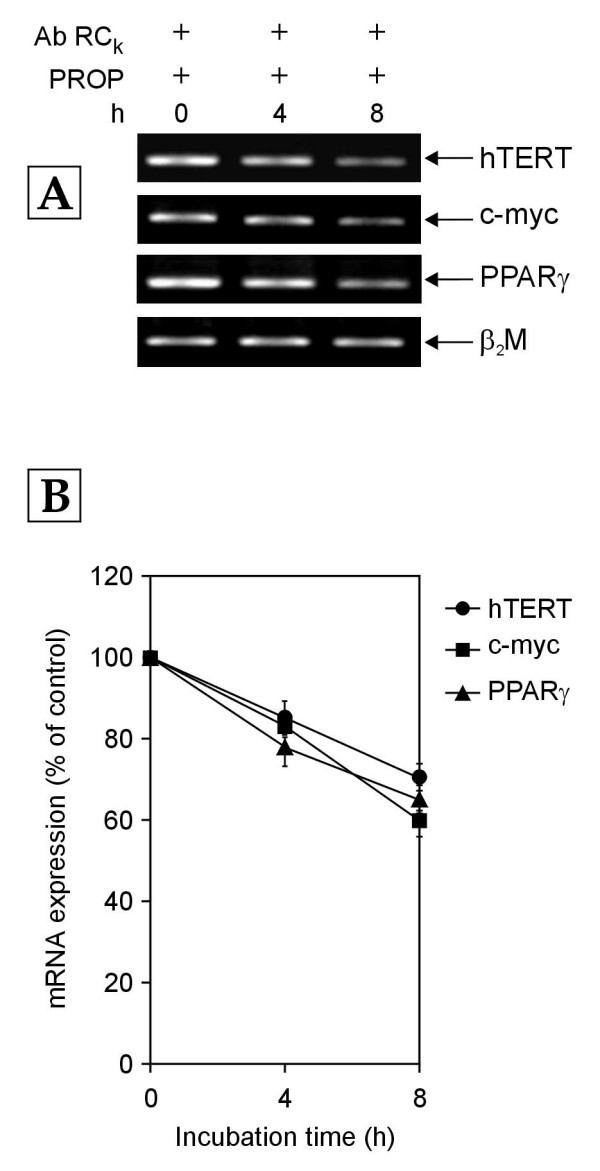
**RT-PCR analysis of expression of hTERT, c-myc and PPARγ mRNA in normal human PBMCs exposed to growth medium containing 10% NHS along with antibody against receptor C_k _(Ab RC_k_) and propranolol (PROP; 200 μM) for 0, 4 and 8 h (hours)**. (A) Representative agarose gel photographs showing ethidium bromide stained RT-PCR products of hTERT, c-myc, PPARγ and β_2_M genes. (B) The signal intensities of RT-PCR products were measured using SCION IMAGE analysis software and the mRNA expression was determined by normalizing the band intensity of target mRNA (hTERT, c-myc and PPARγ) to β_2_M band intensity. The normalized mRNA expression of each gene is expressed as percentage of that in control (0 h) cells. Each data point represents mean ± SD of three independent experiments.

To further verify the involvement of PKC in the Receptor C_k _signaling pathway, the effect of PKC inhibitor was investigated in a situation where Receptor C_k _activation was blocked. The normal human PBMCs were cultured in growth medium enriched with 10% NHS in the presence of antibody against Receptor C_k _and 200 μM of propranolol. Propranolol is known to be an inhibitor of the enzyme phosphatidic acid phosphohydrolase (PAP), which catalyses the conversion of PA to DAG [29]. Hence, propranolol indirectly inhibits PKC by blocking the generation of its activator, DAG from PA. The addition of propranolol abrogated the increase in mRNA expression of c-myc, PPARγ and hTERT genes that was observed in PBMCs exposed to growth medium containing 10% NHS and antibody against Receptor C_k _(Figures 2 &9). In the PBMCs exposed to antibody against Receptor C_k _along with propranolol, the levels of c-myc, PPARγ and hTERT mRNA slightly decreased over 8 hours (Figure 9). This observation suggests that the upregulation of the transcription of c-myc, PPARγ and hTERT genes in PBMCs exposed to antibody against Receptor C_k _alone was due to PKC activation because the upregulation was abolished by PKC inhibition brought about by the addition of propranolol. Hence, these data support the notion that the down-regulation of the transcription of c-myc, PPARγ and hTERT genes by activated Receptor C_k _(Figure 1) involves PKC inhibition while the upregulation of the transcription of these genes observed during the blockage of Receptor C_k _activation (Figure 2) involves the activation of PKC.

### Contribution of PKC to the 25OH-C mediated biphasic effect on the transcription of hTERT, c-myc and PPARγ genes

**Figure 10:**
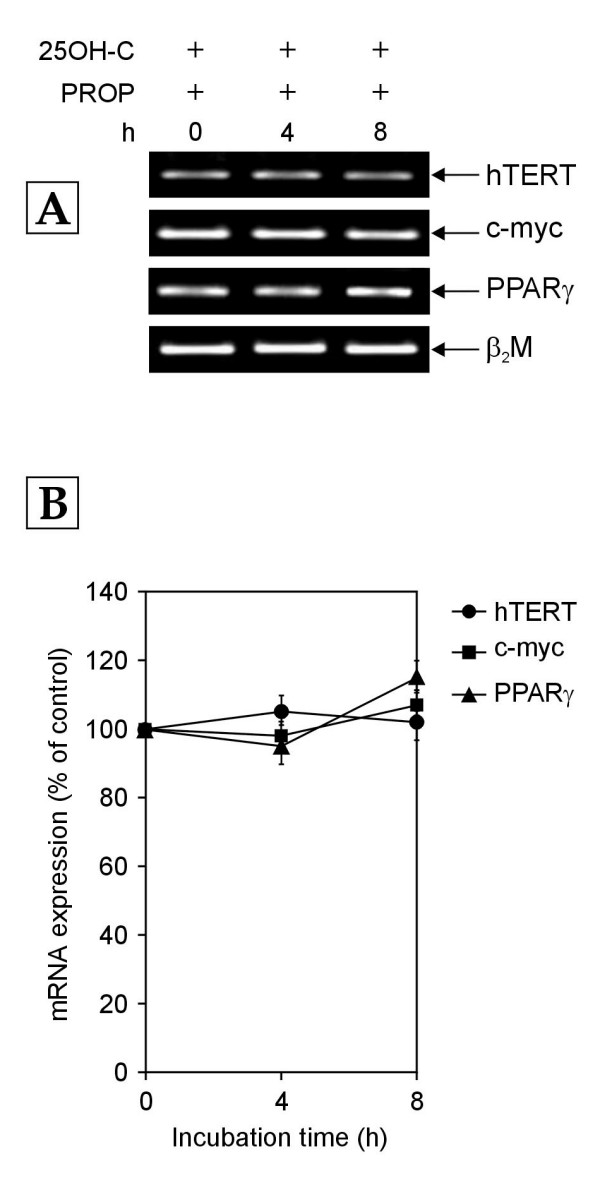
**The mRNA expression of hTERT, c-myc and PPARγ genes in normal human PBMCs treated with growth medium containing 10% NHS and 25-hydroxycholesterol (25OH-C; 1 μM) along with propranolol (PROP; 200 μM)**. Total cellular RNA was extracted at indicated time points (h-hours) and amplified by RT- PCR. (A) Representative agarose gel photographs showing ethidium bromide stained RT-PCR products of hTERT, c-myc, PPARγ and β_2_M genes. (B) The normalized mRNA expression of hTERT, c-myc and PPARγ genes obtained after analysis with SCION IMAGE software is expressed as percentage of that in control (0 h) cells. Each data point represents mean ± SD of three independent experiments.

We also investigated the involvement of PKC in 25OH-C mediated transcriptional regulation of c-myc, PPARγ and hTERT genes. For this, normal human PBMCs were cultured in growth medium containing 10% NHS and 1 μM 25OH-C along with 200 μM of propranolol. As depicted in Figure 10, addition of propranolol completely abrogated the biphasic effect of 25OH-C on the mRNA expression of c-myc, PPARγ and hTERT genes that was observed in PBMCs exposed to medium containing 10% NHS and 1 μM 25OH-C (Figure 5). Since the action of propranolol involves the blocking of the conversion of PA to DAG leading to PKC inhibition, the above observation suggests that the 25OH-C mediated biphasic effect involves activation of PKC.

## Discussion

**Figure 11:**
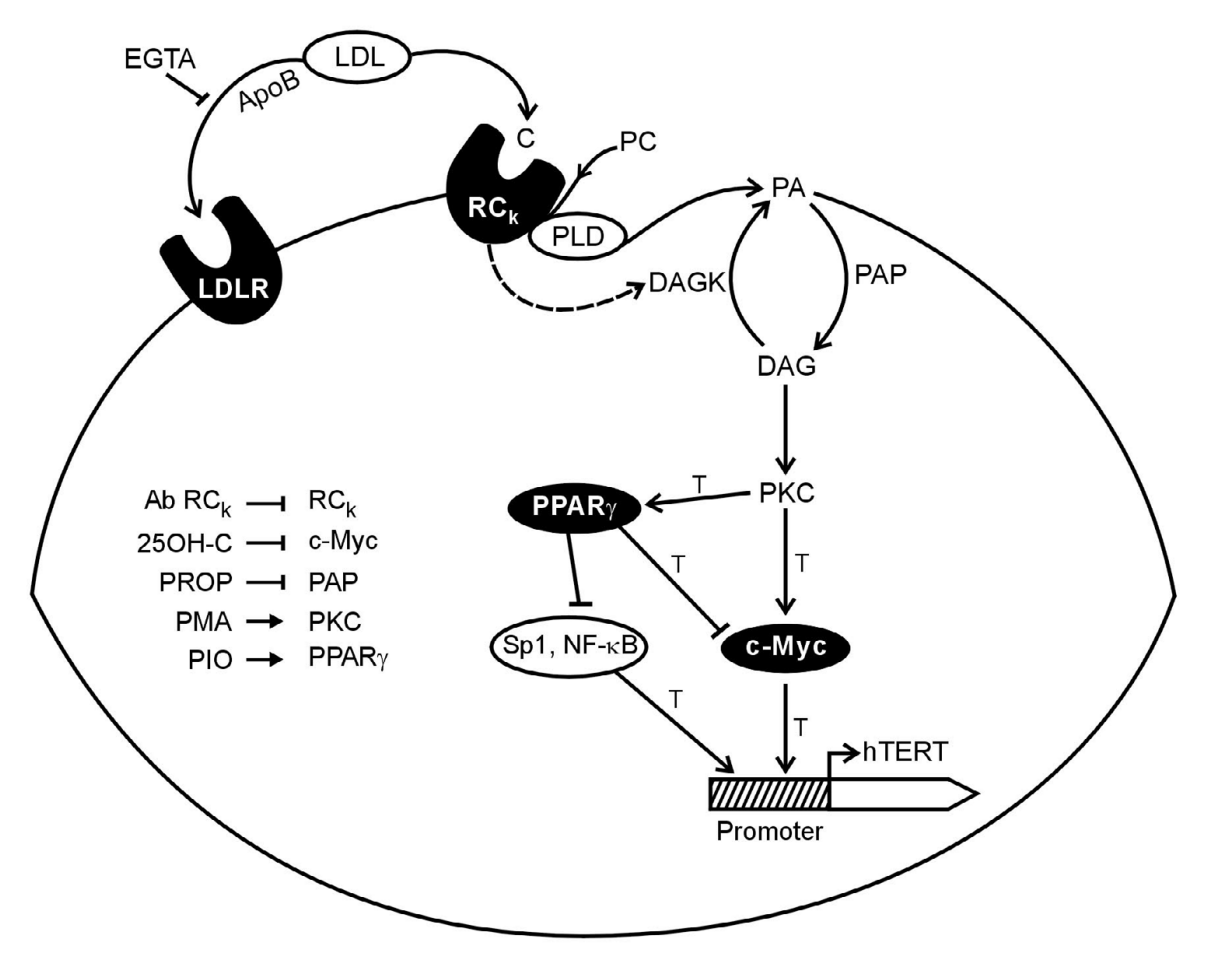
**Proposed Receptor C_k _– dependent signaling pathway involved in hTERT gene regulation at the transcriptional level**. [LDL-low density lipoproteins; C-cholesterol moiety; RC_k_-Receptor C_k_; PC-phosphatidylcholine; PLD-phospholipase D; PA-phosphatidic acid; DAGK-diacylglycerol kinase; PAP-phosphatidic acid phosphohydrolase; DAG-diacylglycerol; PKC-protein kinase C; PPARγ-peroxisome proliferator activated receptor γ; NF-κB-nuclear factor -κB; hTERT-human telomerase reverse transcriptase; T-transcription; LDLR-low density lipoprotein receptor; ApoB-apolipoprotein B; EGTA-ethyleneglycol-bis-(β-aminoethylether)-N,N,N',N'-tetraacetic acid; AbRC_k_-antibody against Receptor C_k_; 25OH-C -25hydroxycholesterol; PROP-propranolol; PMA-phorbol 12-myristate 13-acetate; PIO-pioglitazone]

The present study has identified novel players, namely, cholesterol specific Receptor C_k_, PPARγ and oxysterol (25OH-C) in the transcriptional regulation of hTERT gene. Based on the results of our experiments, we have proposed a signaling pathway (Figure 11) that links Receptor C_k _with hTERT gene transcription through the transcription factors, c-myc and PPARγ. Despite the ability of activated Receptor C_k _to generate the second messenger PA as reported by previous studies, the experiments of this study demonstrate that Receptor C_k _is not able to activate PKC. The data of the present study suggest that activated Receptor C_k _may be involved in the inhibition of PKC. To explain this, it was speculated that Receptor C_k _could inhibit PKC by blocking the production of DAG either by inhibiting the enzyme PAP, which catalyses the conversion of PA to DAG or by activating the enzyme diacylglycerol kinase (DAGK) which catalyses the conversion of DAG back to PA. The inhibition of PAP by Receptor C_k _does not explain the time-dependent biphasic effect on the transcription of c-myc, PPARγ and hTERT genes that was observed when normal human PBMCs were exposed to 25OH-C (Figure 5). However, all the results are compatible with the Receptor C_k _dependent DAGK activation model. Hence, we propose that activated Receptor C_k _may be inhibiting PKC by activating DAGK (Figure 11).

The findings of this study reveal, for the first time, a link between cholesterol (through Receptor C_k_) and transcription of hTERT gene in normal human PBMCs. The LDL-cholesterol dependent activation of Receptor C_k _was found to down-regulate hTERT gene transcription by down-regulating the transcription of c-myc gene through PKC inhibition (Figure 11). The Receptor C_k _dependent down-regulation of hTERT transcription observed in normal human PBMCs may reflect a general mechanism for the repression of hTERT and telomerase expression in normal somatic cells. It is attractive to suggest that the failure of this Receptor C_k _dependent repressive mechanism may contribute to upregulation of hTERT transcription and induction of telomerase activity in cancer cells. In this context, it is interesting to note that Receptor C_k _expression has been found to be lacking in several leukemic cell lines / patients [15].

The treatment of cells with PMA (PKC activator) was found to abolish the Receptor C_k _dependent transcriptional down-regulation of hTERT, PPARγ and c-myc genes and result in time-dependent increase in the mRNA levels of these genes (Figures 1 &8). On the other hand, PKC inhibition by propranolol resulted in the down-regulation of mRNA expression of these genes (Figure 9). The upregulation of PPARγ mRNA and protein expression by PKC activators and its down-regulation by PKC inhibitors has been observed by other groups [28,30,31]. Also, the upregulation of c-myc gene expression by activation of PKC has been reported [32]. Several studies have suggested the important role of PKC isoforms in the upregulation of telomerase activity through the PKC-mediated phosphorylation of hTERT protein [7]. However, the role of PKC in the transcription of hTERT gene has not been thoroughly investigated. Kim et al. [33] observed that PKC inhibitors decreased telomerase activities by decreasing the expression of full length hTERT transcripts in human cervical cancers. Consistent with this, the results of the present study also suggest the involvement of PKC inhibition in the down-regulation of hTERT gene transcription and PKC activation in the upregulation of hTERT gene transcription.

The activation of PPARγ by its agonist pioglitazone was found to down-regulate the mRNA expression of c-myc and hTERT genes in PBMCs (Figure 6). Also, hTERT mRNA expression was upregulated in PBMCs in which PPARγ mRNA expression was knocked down as compared to that in control PBMCs (Figure 7). This is, to the best of our knowledge, the first report of a link between PPARγ activation and hTERT gene transcription. The inhibition of c-myc mRNA expression by activated PPARγ has also been reported by other groups. Activation of PPARγ by ligands such as troglitazone and 15-deoxy-Δ^12,14 ^– prostaglandin J_2 _has been shown to down-regulate c-myc mRNA and protein expression in colon cancer cells and leukemic cell lines [34,35]. Whether PPARγ downregulates hTERT gene transcription through a c-myc dependent or c-myc independent mechanism remains an open question. It has been reported that PPARγ directly interacts with the transcription factor Sp1 at the protein level leading to inhibition of Sp1 and thereby, the transcriptional down-regulation of Sp1 target genes [36,37]. Also, activated PPARγ has been shown to antagonize the activity of the pleiotropic transcription factor, nuclear factor kappa B (NF – κB) thus inhibiting the transcription of NF-κB target genes [38,39]. Both of these transcription factors (Sp1 and NF-κB) have been found to positively regulate hTERT transcription [7-9]. Hence, the effect of activated PPARγ on hTERT mRNA expression may also be mediated by the transcription factors Sp1 and NF-κB (Figure 11). The possibility of a direct effect of activated PPARγ on hTERT gene transcription through a PPARγ specific binding site on hTERT promoter remains to be explored.

The link between activated PPARγ and hTERT transcription observed in this study suggests that PPARγ activation may have important implications in the processes of carcinogenesis and ageing. Recent studies have implicated PPARγ in the regulation of genes relevant to carcinogenesis [20]. Deficient expression of PPARγ can be a significant risk factor for carcinogenesis, although in some cases, PPARγ overexpression has been demonstrated to enhance carcinogenesis [20]. Our finding that PPARγ negatively regulates hTERT transcription suggests that deficient expression of PPARγ may contribute to carcinogenesis through the upregulation of hTERT expression. The link between PPARγ and hTERT gene expression also assumes importance in the light of the fact that PPARγ can be activated by a large number of ligands, which include polyunsaturated fatty acids, metabolites of prostaglandin J, antidiabetic drugs (thiazolidinediones) and a variety of nonsteroidal anti-inflammatory drugs [40]. All these molecules may repress hTERT gene expression and thereby, telomerase activity through PPARγ activation. It is interesting to note here that ligand activation of PPARγ has been shown to inhibit proliferation and induce apoptosis in several cancer cells such as, breast, colon and prostate cancer cells and in various leukemic cell lines [34,35].

Besides implicating cholesterol (through Receptor C_k_) in the transcriptional regulation of hTERT gene, the present study reveals the involvement of oxysterols, the oxygenated derivatives of cholesterol, in the regulation of hTERT gene transcription. Initially used in the study as an inhibitor of c-myc mRNA expression, the oxysterol, 25OH-C was found to have a dual effect on the expression of hTERT gene. This dual effect was found to depend on the activation / inactivation of Receptor C_k_. When Receptor C_k _was not activated due to the presence of antibody against Receptor C_k _in the 'PBMCs + Medium + 10% NHS + 1 μM 25OH-C + Antibody against Receptor C_k_' culture system, 25OH-C repressed hTERT mRNA levels by inhibiting c-myc mRNA expression (Figure 4). In contrast, when 25OH-C was present along with activated Receptor C_k _as in 'PBMCs + Medium + 10% NHS + 1 μM 25OH-C' culture experiment (Figure 5), it abrogated the Receptor C_k _dependent down-regulation of hTERT, c-myc and PPARγ mRNA expression and transiently upregulated the mRNA levels of these genes (Figures 1 &5). A time-dependent biphasic effect on the mRNA levels of hTERT, c-myc and PPARγ genes was observed in this situation (Figure 5) suggesting that 25OH-C might be interacting at some point with the Receptor C_k _signaling pathway, leading to the transient upregulation of the genes under study. In an attempt to understand the mechanism underlying the observed biphasic effect of 25OH-C, we investigated the effect of PKC inhibition on this action of 25OH-C. Inhibition of PKC by propranolol was found to abolish the biphasic effect of 25OH-C (Figure 10) showing that the 25OH-C mediated transient disruption of Receptor C_k _dependent signaling involved the conversion of PA to DAG and PKC activation. This observation coupled with the previous report that 25OH-C possesses the ability to amplify the Receptor C_k _dependent generation of PA [42] suggests that PA generation followed by the conversion of PA to DAG and PKC activation may be the point at which 25OH-C interacts with Receptor C_k _dependent signaling pathway. Due to the increase in the generation of PA when 25OH-C is present along with activated Receptor C_k_, PKC could be activated (even in the presence of activated Receptor C_k_) (Figure 11), leading to the observed transient upregulation of the mRNA expression of c-myc, PPARγ and hTERT genes (Figure 5). Decrease in the mRNA levels of these genes, observed during the biphasic effect is due to the Receptor C_k _dependent DAGK activation resulting in the inhibition of PKC (Figures 5 &11). Hence, the biphasic effect on the mRNA expression of c-myc, PPARγ and hTERT genes observed when 25OH-C is present along with activated Receptor C_k _is attributed to the ability of 25OH-C to activate PKC and the ability of activated Receptor C_k _to inhibit PKC (Figures 5 &11). These observations, which suggest a role for the oxygenated derivative of cholesterol in the regulation of hTERT gene transcription and more importantly, reveal the ability of 25OH-C to reverse the Receptor C_k _dependent transcriptional repression of hTERT, c-myc and PPARγ genes may have important implications given the diverse biological roles of oxysterols. It is pertinent to note here that 25OH-C is one of the abundant oxysterols present in the human plasma [43,44]. The oxysterols have been implicated in a variety of cellular processes including lipid metabolism, apoptosis and cell differentiation [43]. Several studies have indicated that oxysterols play an important role in the development of atherosclerosis [43]. Oxysterols have also been suggested as potential cancer chemotherapeutic agents [43].

The peripheral blood immune cells exhibit particularly interesting telomere dynamics. Despite the presence of telomerase, the telomeres in these cells shorten progressively with increasing age and with cellular replication in vitro [45,46]. Telomere attrition even in the presence of telomerase suggests that the level of hTERT and telomerase expression is insufficient for the maintenance of telomere lengths. This deficient expression of hTERT and telomerase in PBMCs possibly represents a tumor suppressor mechanism, essential for the protection of these cells against the development of cancer. The Receptor C_k_-dependent down-regulation of hTERT mRNA expression observed in the present study may cooperate with the other repressive mechanisms operational in PBMCs in order to maintain appropriate low levels of telomerase in these cells. Further, it has been reported that unlike most telomerase positive cells, peripheral blood lymphocytes do not exhibit a tight correlation between hTERT mRNA expression and telomerase activity. The hTERT transcripts are expressed in these cells independently of the presence, absence or quantitative level of detectable telomerase activity [47]. However, hTERT transcription is upregulated along with the increase in telomerase activity during the activation of peripheral blood cells. [47]. In the present study, since Receptor C_k _activation has been found to down-regulate hTERT transcription, it is expected to repress telomerase activity as well, because the enzymatic activity of telomerase can not possibly occur in the absence of hTERT, the catalytic subunit of telomerase. The absence of correlation between hTERT mRNA expression and telomerase activity in PBMCs suggests that hTERT may have some other roles besides contributing to telomerase activity and telomere lengthening. Recent studies have, indeed, shown that actions of hTERT in cellular proliferation and tumor progression extend beyond the singular role of telomere maintenance [48]. hTERT has also been shown to enhance cell survival independent of its effect on telomere length maintenance [48]. Hence, the study of hTERT transcription irrespective of its contribution to telomerase activity assumes importance. Besides participating in the suppression of telomerase, Receptor C_k _dependent down-regulation of hTERT transcription may be involved in the other as yet uncharacterized roles of hTERT in PBMCs.

## Conclusion

The findings of this study provide new insights into the regulation of hTERT transcription and may have important implications in cancer and ageing research.

## Methods

### Materials

Lipoprotein deficient serum (LPDS), fetal bovine serum (FBS), Ethyleneglycol-bis-(β-aminoethylether)-N,N,N',N' -tetraacetic acid (EGTA), 25-Hydroxycholesterol (25OH-C), Phorbol 12-myristate 13-acetate (PMA) and DL-Propranolol hydrochloride were purchased from Sigma (U.S.A.). Pioglitazone was kindly provided by Dr. S. Varma (Department of Internal Medicine, Postgraduate Institute of Medical Education & Research, Chandigarh, India). Specific antibodies against cyclin D and Bcl-2 were obtained from Santa Cruz Biotechnology, Inc. (U.S.A.). Polyclonal monospecific antibody against Receptor C_k _was raised in our laboratory as reported earlier [49]. All the other reagents used in the study were of the highest quality commercially available.

### Cell culture and treatments

Normal human peripheral blood mononuclear cells (PBMCs) were obtained from healthy volunteers, who were fasting for 12 hours and abstained from any medication for 2 weeks before blood donation. Blood was drawn through venipuncture into heparinized tubes and PBMCs were isolated using Ficoll-Hypaque gradient centrifugation [50]. These PBMCs were washed twice with phosphate buffered saline and then cultured at a density of 0.5 × 10^6 ^cells/ml in Dulbecco's Modified Eagle's Medium (DMEM) supplemented with 10% LPDS, 2 mM L-glutamine, 20 mM HEPES (N-2-hydroxyethyl piperazine-N' -2-ethanesulphonic acid), 24 mM sodium bicarbonate, 50 units/ml penicillin and 50 μg/ml streptomycin. Culturing was done for 24 hours at 37°C in humidified 5% CO_2 _atmosphere. At the end of this incubation period, the PBMCs were exposed to fresh DMEM containing 10% heat-inactivated normal human serum (NHS) in place of LPDS. At this stage, PBMCs were cultured for an additional 0, 4, 8 and 12 hours either in the presence of medium + 10% NHS alone or in combination with antibody against Receptor C_k_, EGTA (2.5 mM), 25OH-C (1 μM), PMA (100 nM) or propranolol (200 μM). Cells were harvested by centrifugation at 250 × *g *for 15 minutes and the obtained pellets were subjected to RNA and protein extraction.

To study the effect of PPARγ activation, PBMCs cultured in DMEM containing 10% FBS were used instead of LPDS treated PBMCs. These cells were preincubated with different concentrations (0–50 μM) of pioglitazone (a synthetic PPARγ agonist belonging to the class of thiazolidinedione antidiabetic agents) in FBS-free medium for 4 hours. After 4 hours, 10% FBS was added and the cells were harvested after culturing for additional 20 hours. Harvested cells were subjected to RNA isolation.

Both propranolol and pioglitazone were prepared fresh before use by dissolving in dimethyl sulfoxide (DMSO). Stocks of PMA and 25OH-C were made by dissolving in DMSO and absolute ethanol respectively. These stock preparations were stored at -20°C and diluted immediately before use. Vehicle concentration in the medium was maintained at 0.1% vol/vol. Viable cells always exceeded 90% as determined by trypan blue exclusion dye test.

### RNA extraction

Total cellular RNA was extracted by the acid guanidinium phenol chloroform method [51]. RNA yield and purity were determined spectrophotometrically at 260–280 nm and the integrity of RNA verified by electrophoresis through denaturing agarose gels stained with ethidium bromide.

### Determination of c-myc, hTERT and PPARγ mRNA expression by reverse transcriptase-polymerase chain reaction (RT-PCR)

First strand cDNA was synthesized from total extracted RNA using the RevertAid™ first strand cDNA synthesis kit from Fermentas. The cDNA was then amplified using primers specific for c-myc, hTERT, PPARγ and β_2 _microglobulin (β_2_M) under the conditions described elsewhere [52-55]. PCR was performed using 24, 36, 24 and 17 cycles for c-myc, hTERT, PPARγ and β_2_M amplification respectively. The number of cycles was determined in preliminary experiments to be within the exponential range of PCR amplification. β_2_M expression was used as a control for RNA loading and efficiency of reverse transcription.

PCR products were resolved on 2% agarose gels stained with ethidium bromide. The gels were visualized in ultraviolet light and photographed. Band intensities were evaluated using the SCION IMAGE analysis software. The relative levels of c-myc, hTERT and PPARγ mRNA expression were obtained by the ratio of their individual band intensity to β_2_M band intensity.

### Determination of cyclin D and Bcl-2 expression by western blotting

Equal amounts of proteins per sample were separated on 12.5% sodium dodecyl sulphate – polyacrylamide gels and then electro-transferred to nitrocellulose membranes. The membranes were probed using specific antibodies against cyclin D and Bcl-2.

### Silencing of PPARγ gene using small interfering RNA (siRNA)

Total cellular RNA was extracted from normal human PBMCs and subjected to RT-PCR using primers specific for PPARγ [52]. This PCR product was employed as a template for the production of double stranded RNA (dsRNA) using the BLOCK-iT™ RNAi TOPO Transcription kit (Invitrogen). The dsRNA was diced into PPARγ gene-specific siRNA using the BLOCK-iT™ Dicer RNAi kit (Invitrogen). Scrambled siRNA was used as a negative control. PPARγ-specific siRNA or scrambled siRNA were transfected into normal human PBMCs using the Lipofectamine™ 2000 reagent (Invitrogen). These cells were harvested after 72 hours and subjected to RNA extraction and RT-PCR analysis in order to determine the relative levels of PPARγ and hTERT mRNA expression.

## Authors' contributions

KS participated in the design of the study, carried out all the experiments and drafted the manuscript. DK conceived of the study, provided valuable insights into the interpretation of results and helped draft the manuscript. NV helped in hematological analysis of the volunteers whose blood mononuclear cells were used in this study.
